# Supplementary material for: Replicating shear-mediated self-assembly of spider silk through microfluidics
Source: Nat Commun. 2024 Jan 15;15:527. doi: 10.1038/s41467-024-44733-1 (PMC10789810; doi:10.1038/s41467-024-44733-1)
Supplement: Supplementary file 1 — Supplementary Information [file 41467_2024_44733_MOESM1_ESM.pdf]

Supplementary Information

Replicating shear-mediated self-assembly of spider silk through microfluidics

Jianming Chen,[1,2,3,4] Arata Tsuchida,[5] Ali D. Malay,[1] Kousuke Tsuchiya,[6] Hiroyasu Masunaga,[7] Yui Tsuji,[6] Mako Kuzumoto,[6] Kenji Urayama,[6] Hirofumi Shintaku,[6] and Keiji Numata*[1,6,8]

[1] Biomacromolecules Research Team, RIKEN Center for Sustainable Resource Science, 2-1 Hirosawa, Wako, Saitama 351-0198, Japan.

[2] Research Institute for Intelligent Wearable Systems, The Hong Kong Polytechnic University, Kowloon, Hong Kong.

[3] Research Centre of Textiles for Future Fashion, The Hong Kong Polytechnic University, Kowloon, Hong Kong.

[4] School of Fashion and Textiles, The Hong Kong Polytechnic University, Kowloon, Hong Kong.

[5] Cluster for Pioneering Research, RIKEN, 2-1 Hirosawa, Wako, Saitama 351-0198, Japan.

[6] Department of Material Chemistry, Kyoto University, Nishikyo-ku, Kyoto 615-8510, Japan.

[7] Japan Synchrotron Radiation Research Institute, 1-1-1, Kouto, Sayo-cho, Sayo-gun, Hyogo 679-5198, Japan.

[8] Institute for Advanced Biosciences, Keio University, Tsuruoka, Yamagata, 997-0017, Japan.

* Correspondence to K.N. (email: keiji.numata@riken.jp)

Supplementary Discussion

Rationale of microfluidic design

Our aim was to develop a platform that incorporates different physiochemical triggers that are involved in the natural spider silk spinning system into a single microfluidic device and use this device to drive the self-assembly of recombinant MaSp2 fibers with native-like hierarchical structures. From a chemical standpoint, the physiochemical triggers include an ionic gradient mimicking the transition from a chaotropic (chloride-enriched) to a kosmotropic (phosphate-enriched) aqueous environment along the spinning ducts[1] and a pH gradient mimicking the well-studied acidification process in the spinning ducts, which is crucial for silk fiber formation (with the pH gradient estimated to range from approximately pH 7.2 in the protein storage sac down to approximately pH 5.7, or considerably lower, in the distal parts of the spinning duct of the spider major ampullate gland)[2]. Dehydration (the exclusion of residual water from the protein material) is also important in the native silk spinning process[3]; in our system, this water removal was mimicked by the presence of a salt gradient, further promoting the phase separation of proteins. The design of the microfluidic device itself, particularly the dimensions and geometries of different channels, is of course a major consideration, as these geometric parameters are inextricably linked to the flow behavior of the liquid spinning dope and have remarkable impacts on the elongational flow, shear forces, etc., applied to the polypeptide chains and their consequent effects on chain alignment, as well as the balance among different chemical gradients that act simultaneously inside the spinning duct. In this regard, the internal geometry of the spinning duct of the major ampullate gland has been characterized as having an initial section with an essential shape of a hyperbolic curve followed by an extended section with a linearly tapering diameter[4]. For the purposes of the current study, however, the desire to achieve an ideal biomimetic chip design was counterbalanced by the practical need to create a system that was both easy to manufacture and operate and simple enough to computationally model.

Although a series of microfluidic devices were developed to prepare artificial spider silk fibers by mimicking the natural spinning process[5-7], most of them were limited to discussion on the effects of salting-out and decreasing pH by running the microfluidic system with a syringe pump or positive pressure in a manner of extrusion[31,54, 55]. In

this context, a controlled negative pressure system was established to create a shear flow inside the microfluidic chip, allowing the simulation of shear stress and the demonstration of its effect on silk assembly and β-sheet formation. Therefore, under a specific negative pressure, the related shear stress was estimated by modeling to match the β-sheet content calculated from Raman deconvolution for the aim of quantitatively analyzing shear-induced crystallization.

Hierarchical organization in silk fibers

One of the hallmark features of spider silk is its hierarchical structure. As revealed by this study, the hierarchical organization originated from the nanofibrillation induced by salting-out and acidification and later developed by shear force with an orientation along the fiber axis. Although several studies declared that the "hierarchical" structure was reconstructed for artificial silk fibers[8,9], it was different from the native-like structure with regard to the morphology, length scale, and molecular formation mechanism. The current dilemma is that most recombinant spider silk fibers had a much lower mechanical property than natural spider silks, even though a few studies demonstrated that comparable tensile strength or toughness was achieved[7,10]. We envision that largely improved and balanced mechanical behavior can be achieved once artificial silk fibers are equipped with subtle fibrillar ordered organization.

The influence of polyalanine blocks on LLPS and nanofibrillization

It is of great interest to determine the effect of polyalanine blocks on the hierarchical and secondary structures during the silk assembly process. For this purpose, variant N-R12-C(xA) was designed without the polyalanine motif for comparison with wild-type N-R12-C. As expected, polyalanine blocks are responsible for β-sheet formation regardless of which spinning method (e.g., microfluidic spinning or manual stretching) is adopted. It is noteworthy that the morphological change from LLPS and nanofibrillation to solid fiber certainly did not alter protein conformation, which is particularly sensitive to physical forces, such as those under shearing and stretching. Similar to the spinning dope, MaSp2 in the LLPS and nanofibrillation stages retains the helical silk I structure. It remains unclear which kind of amino acid residues cause LLPS and nanofibrillation, although this would be considerably interesting information to know. In our work, we find that alanine residues in the form of seven repeats exhibit negligible influence on these processes. This result is meaningful for

subsequent research, in which the alanine residues can be flexibly engineered to mediate an ideal secondary structure without worrying about sacrificing hierarchical structure.

LLPS as a paradigm for silk self-assembly mechanism

Two theories, namely, liquid crystalline (LC)[11] and micelle theory[12], were proposed approximately twenty years ago to explain the silk assembly mechanism. In this work, native-like silk assembly with MaSp2 was achieved at a relatively low concentration of 50 mg/ml, which did not meet the condition required for LC theory. Although the dynamic fusing behavior of MaSp2 droplets showed certain similarity to the coalescence of micelles, we would rather classify our work as representative of LLPS, an additional paradigm to LC/micelle theories for assembly mechanisms. LLPS and micelle theory may not be exclusive to each other, and the exact mechanism of LLPS remains to be determined. LLPS is even more impressive when considering that it is followed by nanofibrillation, which demonstrates the intimate relationship between the hierarchically organized structures found for natural dragline silk and that of our MaSp2 fiber. It is fascinating to further explore LLPS due to its great potential in the following aspects. In place of the traditional method using dialysis against polyethylene glycol (PEG) solution, LLPS can be used as an effective tool to concentrate spidroins by introducing multivalent anions, such as phosphate, citrate and sulfate ions. Additionally, LLPS can also be developed as a promising protocol for directly purifying target spidroins from cells in the absence of His-tags in comparison with conventional immobilized metal affinity chromatography (IMAC)[13].

Supplementary Figures

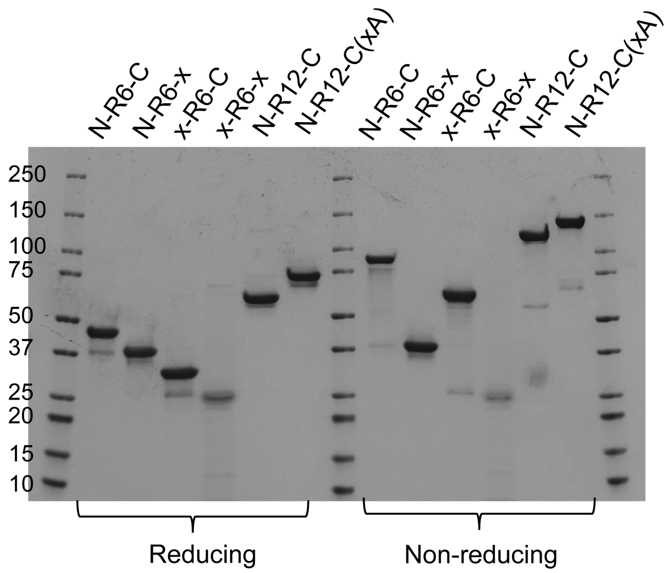

Supplementary Fig. 1: Expression of recombinant MaSp2 as demonstrated by SDS–PAGE. In the presence of the C-terminus, MaSp2 formed dimers in solution. However, by adding the reducing agent (2-mercaptoethanol), these MaSp2, such as NR_6C, R_6C, $NR_{12}C$ and $NR_{12}C$-xA, only existed as monomers. Three independent experiments were performed with similar results.

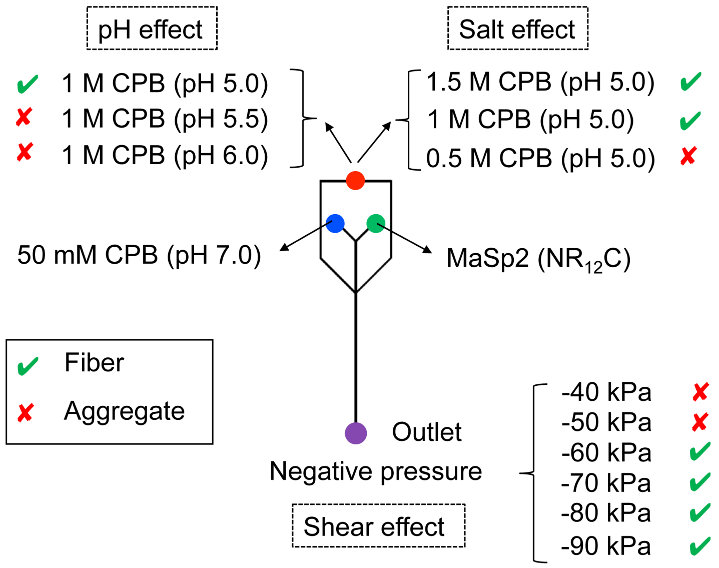

Supplementary Fig. 2: Effects of pH, salt and shear on the morphologies of $NR_{12}C$ inside the microfluidic device. The pH varied from 5.0 to 6.0, and the salt concentration was varied from 0.5 to 1.5 M. The shear effect was triggered by changing the pressures from -40 to -90 kPa. Five independent experiments were performed with similar results.

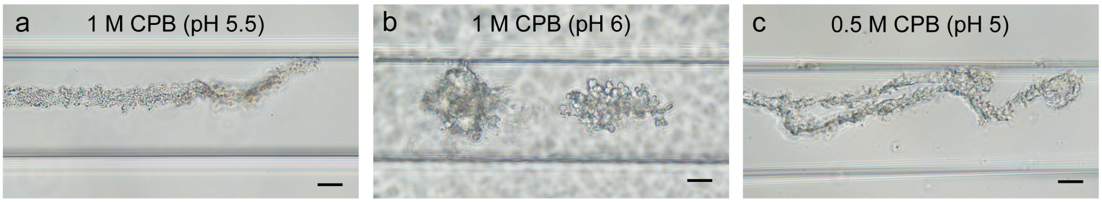

Supplementary Fig. 3: N-R12-C aggregates formed within the microfluidic channel by changing the pH and salt conditions. a and **b,** N-R12-C aggregates formed at pH 5.5 and 6, respectively, without sufficient acidification. **c**, N-R12-C aggregates formed in 0.5 M CPB without sufficient dehydration. Four independent experiments were performed with similar results. Scale bar: 20 μm.

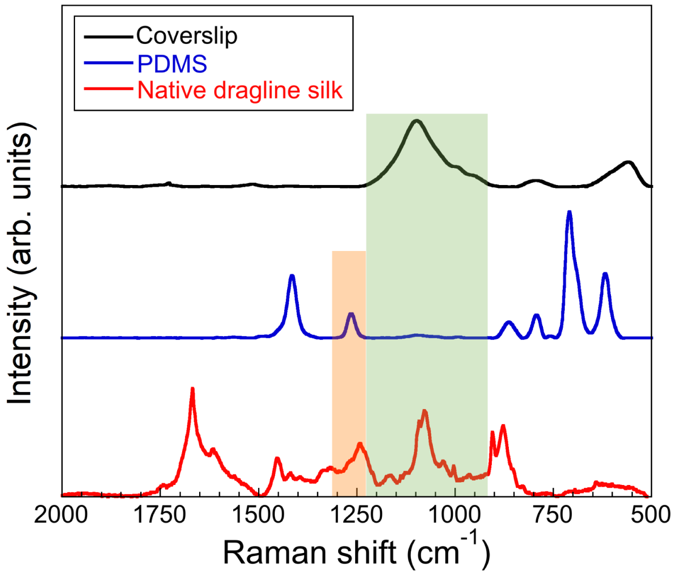

Supplementary Fig. 4: Raman signal shift of native spider dragline silk against the microfluidic background. Cover slides and PDMS showed overlapping signals in specific regions, which are widely used to analyze the secondary structure of spidroins. Therefore, the amide I region was adopted in this work for analysis and comparison.

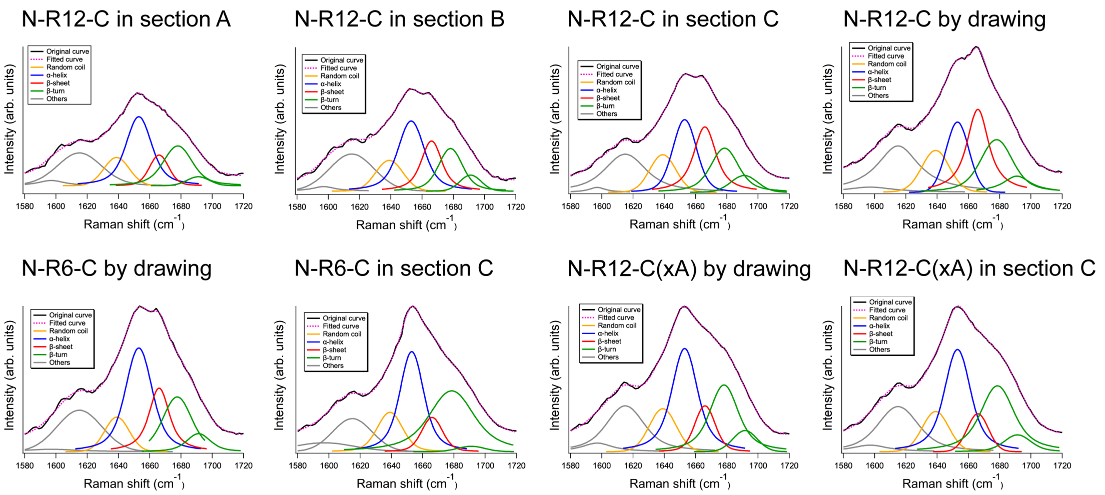

Supplementary Fig. 5: Peak fitting results of various MaSp2 types after deconvolution within the amide I region. The component ratios of the secondary structure could be calculated from the fitting results to show the difference in β-sheet contents from section A to section C for different protein types by two spinning methods.

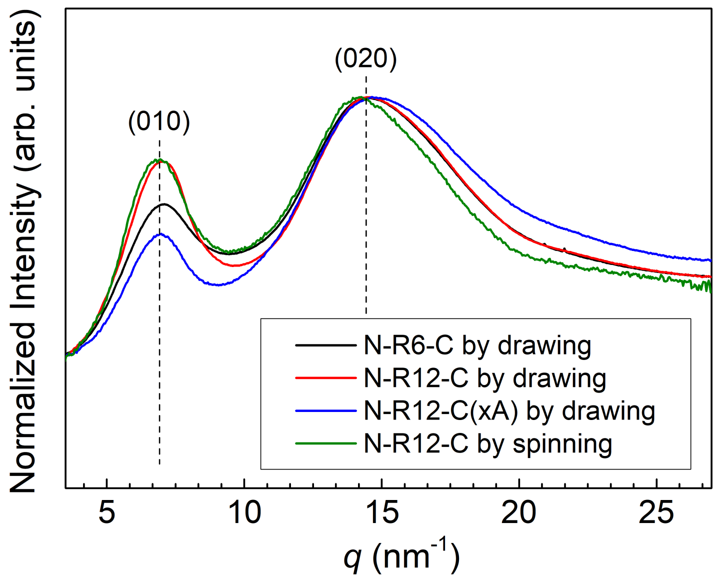

Supplementary Fig. 6: Synchrotron-radiated wide-angle X-ray diffraction (WAXD) results of MaSp2 fibers prepared by microfluidic spinning and manual drawing.

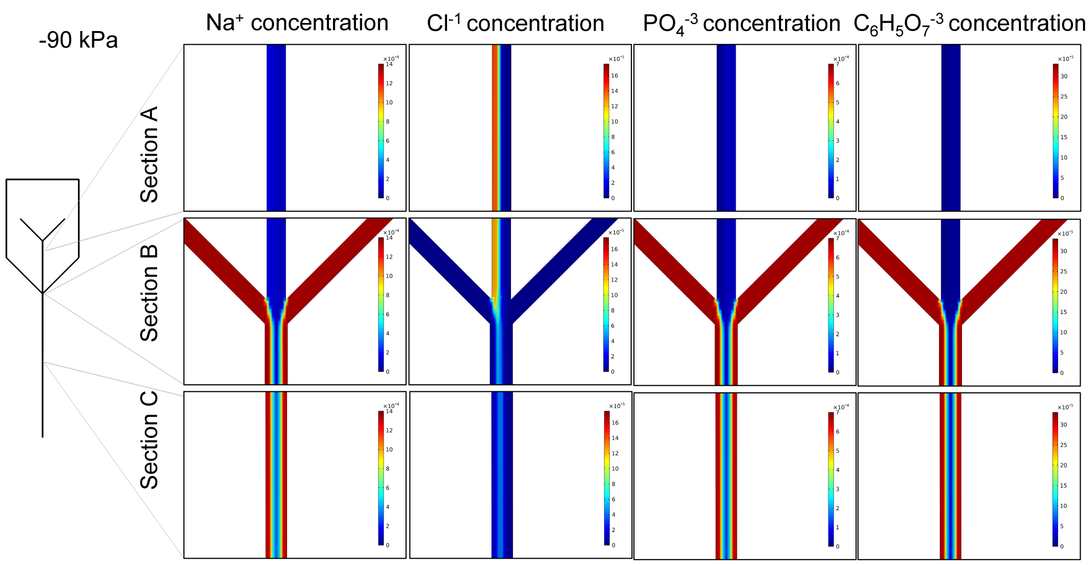

Supplementary Fig. 7: Simulation results of various ionic concentrations along the channel under -90 kPa. These ions were simulated in the longitudinal direction to show the difference from section A to C.

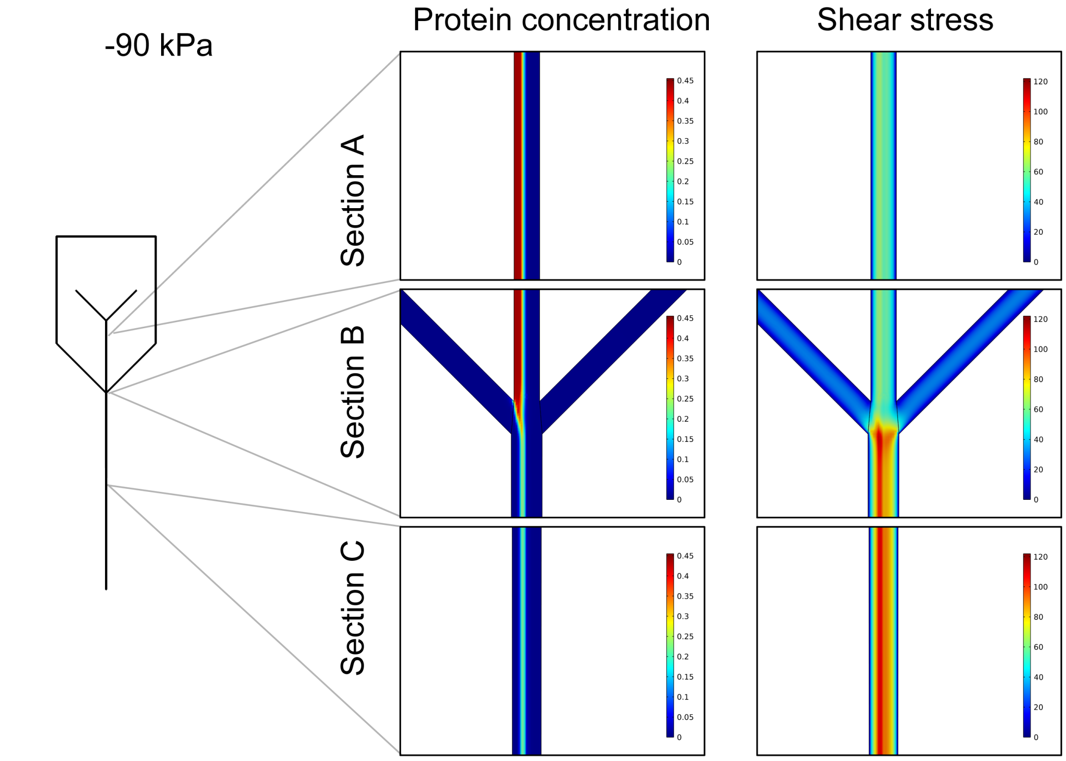

Supplementary Fig. 8: Simulation results of N-R12-C protein concentration, shear rate and stress along the channel under -90 kPa. The protein concentration and shear were simulated in the longitudinal direction to show the difference from section A to C.

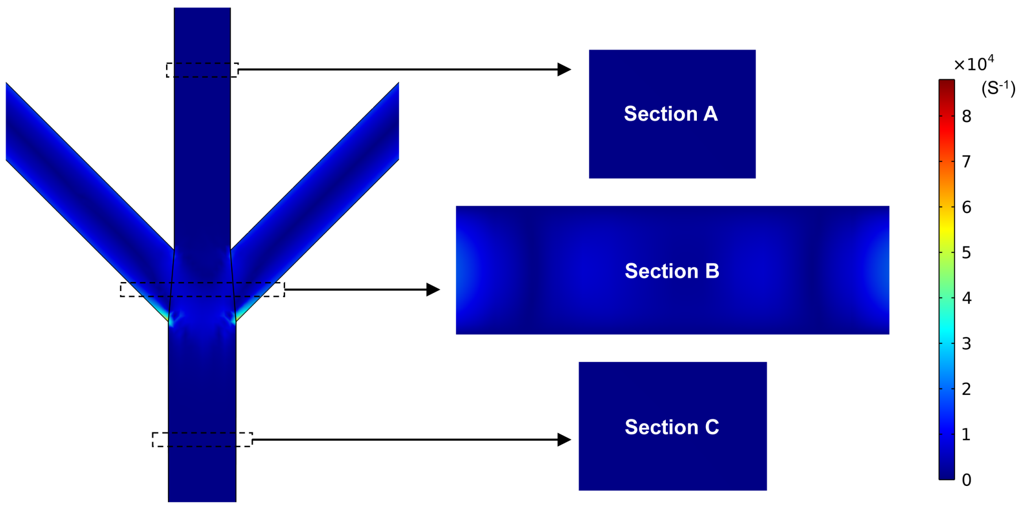

Supplementary Fig. 9: Simulation results of the elongation rate for N-R12-C along the channel under -90 kPa. The elongation rate of N-R12-C was simulated in the longitudinal and cross-section directions to show the difference from section A to C.

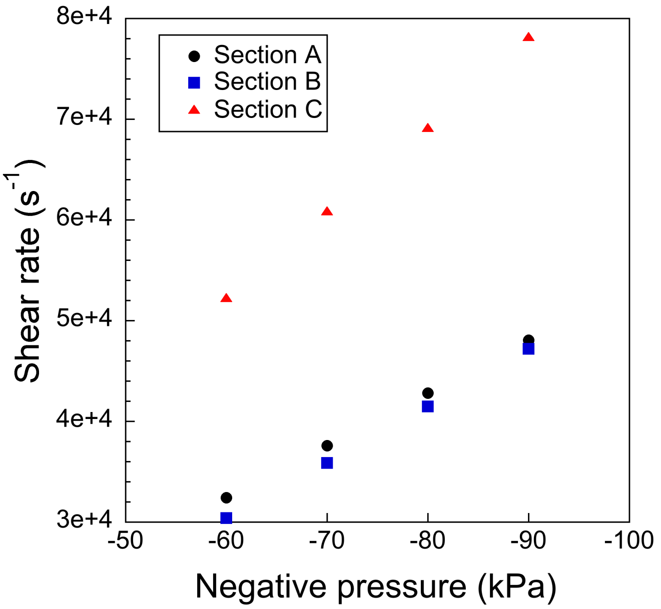

Supplementary Fig. 10: Shear rate simulated for N-R12-C in three sections under negative pressure from -60 to -90 kPa.

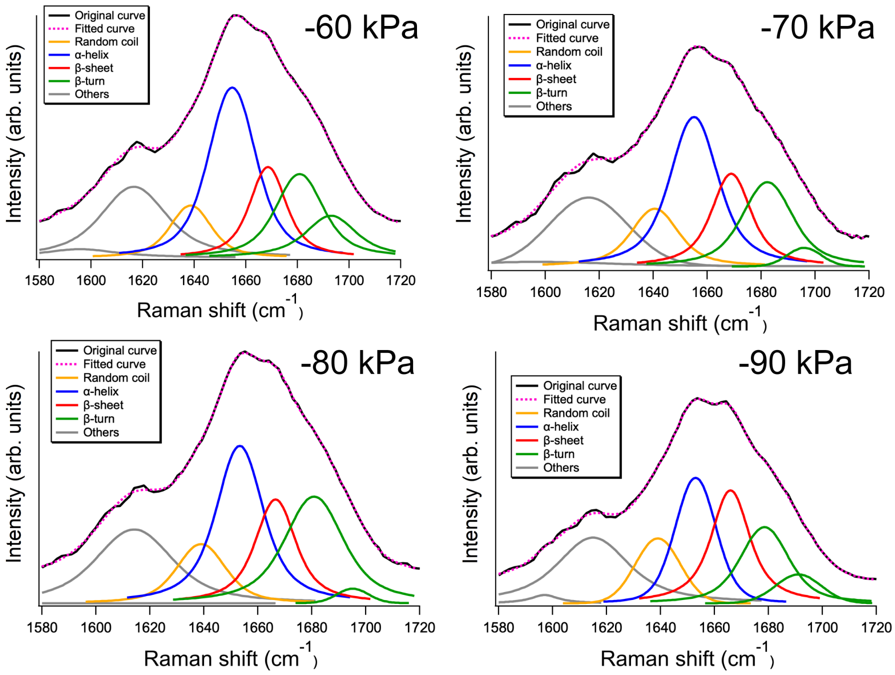

Supplementary Fig. 11: Peak fitting results of N-R12-C fiber formed by biomimetic spinning under various negative pressures. With regard to the increase in pressure from -60 to -90 kPa, the peak at 1664 cm^{-1} became more prominent in comparison to the peak at 1654 cm^{-1}.

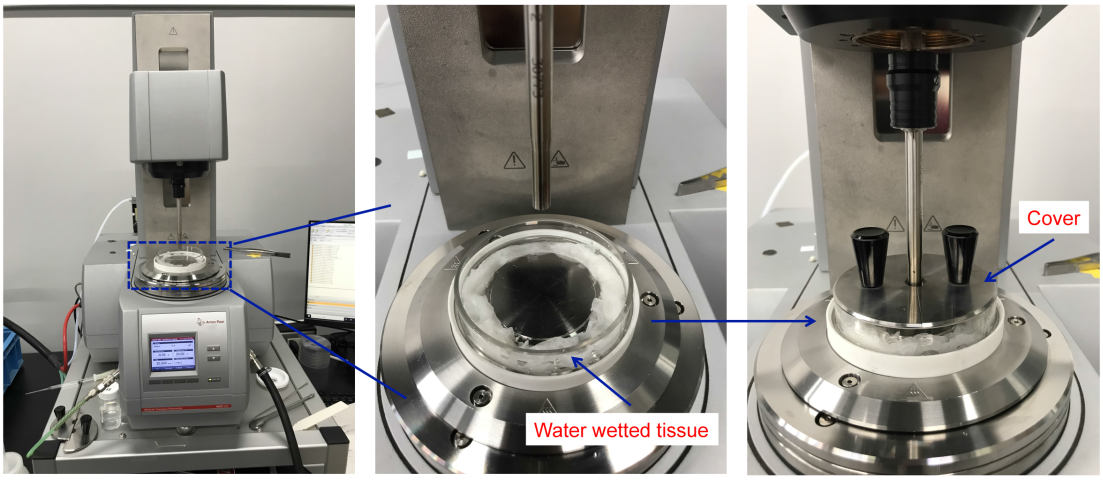

Anton Paar MCR 502 Rheometer

Supplementary Fig. 12: Experimental setup for the MaSp2 (N-R12-C) solution by using Anton Paar MCR 502 Rheometer. The tissue was wetted by the water and placed around the plate without contacting the sample. Finally, a cover was used to maintain saturated humidity of the sample. Three independent experiments were performed with similar results.

Supplementary Tables

Supplementary Table 1. Comparison of difference on the microfluidic spinning of recombinant spidroins

	Type of protein	Domain structure	Flow driving force	Buffer system	pH drop	Fiber formed inside channels	LLPS	Hierarchical structure	Quantification of shear
This work	MaSp2	N-R6-C; N-R12-C	Negative pressure	Citrate phosphate buffer	from 7 to 5	Yes	Yes	Yes	Yes
Rammensee et al[5]	eADF3, eADF4	N-R12-C; N-R'16-C	Syringe pump	Potassium phosphate buffer	from 8 to 6	Yes	ND	No	No
Saric et al[7]	eADF3, eADF4	N-R'16-C; N-R12-C	Syringe pump	Potassium phosphate buffer	from 8 to 6	No	ND	No	No
Saric et al[14]	TIO spidroins; eADF3; eADF4	N-R6-R'8-C; N-R12-C; N-R'16-C	Syringe pump	Potassium phosphate buffer	from 8 to 6	No	ND	No	No
Renberg et al[6]	MaSp1	R4-C; N-R4-C	Positive pressure	Oil	No	No	No	No	No
Chen et al[15]	eTuSp1	R1	Syringe pump	Isopropanol solution	No	No	No	No	No
Peng et al[16]	MaSp2	R16	Syringe pump	Ethanol solution	No	No	No	No	No

ND: Not determined/discussed

Supplementary Table 2. Component ratios of the secondary structure after deconvolution within the amide I region

Sample	Random coil (%)	α-helix (%)	β-sheet (%)	β-turn (%)
N-R12-C in section A	13.9	42.8	12.5	30.8
N-R12-C in section B	13.9	37.8	22.5	25.8
N-R12-C in section C	14.3	27.7	29.2	28.8
N-R6-C in section C	13.1	34.5	9.3	43.1
N-R12-C(xA) in section C	12.3	39.0	9.2	39.5
N-R12-C by drawing	14.6	19.8	32.2	33.4
N-R6-C by drawing	10.0	40.6	21.0	28.4
N-R12-C(xA) by drawing	13.4	39.8	12.1	34.7

Supplementary Table 3. Parameters used in the numerical model by using COSMOL[17]

Parameter	Value
Viscosity of 1 M CPB (pH 5) (μ_{CPB})	1.2×10^{-3} Pa s
a_{inf}	1.4×10^{-1}
a_o	2.9×10^{4}
k_0	2.6
a_λ	3.4
a_n	2.1×10^{-1}
k_n	7.2×10^{-2}
Diffusion coefficient of sodium ion*	1.3×10^{-9} m^2 s^{-1}
Diffusion coefficient of chloride ion*	2.0×10^{-9} m^2 s^{-1}
Diffusion coefficient of phosphate ion*	1.0×10^{-9} m^2 s^{-1}
Diffusion coefficient of citrate ion*	0.6×10^{-9} m^2 s^{-1}
Diffusion coefficient of the silk protein	16.3×10^{-12} m^2 s^{-1}
Absolute temperature	298.15 K

* Diffusion coefficient of ions[18,19]

Supplementary References

1 Knight, D. P. & Vollrath, F. Changes in element composition along the spinning duct in a *Nephila* spider. *Naturwissenschaften* **88**, 179-182 (2001).

2 Andersson, M. *et al.* Carbonic anhydrase generates CO2 and H+ that drive spider silk formation via opposite effects on the terminal domains. *PLoS biology* **12**, e1001921 (2014).

3 Kojic, N., Kojic, M., Gudlavalleti, S. & McKinley, G. Solvent removal during synthetic and Nephila fiber spinning. *Biomacromolecules* **5**, 1698–1707 (2004).

4 Davies, G. J., Knight, D. P. & Vollrath, F. Chitin in the silk gland ducts of the spider Nephila edulis and the silkworm Bombyx mori. *PloS one* **8**, e73225 (2013).

5 Rammensee, S., Slotta, U., Scheibel, T. & Bausch, A. Assembly mechanism of recombinant spider silk proteins. *Proceedings of the National Academy of Sciences* **105**, 6590–6595 (2008).

6 Renberg, B., Andersson-Svahn, H. & Hedhammar, M. Mimicking silk spinning in a microchip. *Sensors and Actuators B: Chemical* **195**, 404–408 (2014).

7 Saric, M., Eisoldt, L., Döring, V. & Scheibel, T. Interplay of different major ampullate spidroins during assembly and implications for fiber mechanics. *Advanced Materials* **33**, 2006499 (2021).

8 Fan, L., Li, J.-L., Cai, Z. & Wang, X. Bioactive hierarchical silk fibers created by bioinspired self-assembly. *Nature communications* **12**, 1–9 (2021).

9 Li, S. *et al.* Microfluidic silk fibers with aligned hierarchical microstructures. *ACS Biomaterials Science & Engineering* **6**, 2847–2854 (2020).

10 Lin, Z., Deng, Q., Liu, X.-Y. & Yang, D. Engineered Large Spider Eggcase Silk Protein for Strong Artificial Fibers. *Advanced Materials* **25**, 1216-1220 (2013).

11 Vollrath, F. & Knight, D. P. Liquid crystalline spinning of spider silk. *Nature* **410**, 541–548 (2001).

12 Jin, H.-J. & Kaplan, D. L. Mechanism of silk processing in insects and spiders. *Nature* **424**, 1057–1061 (2003).

13 Prince, J. T., McGrath, K. P., DiGirolamo, C. M. & Kaplan, D. L. Construction, cloning, and expression of synthetic genes encoding spider dragline silk. *Biochemistry* **34**, 10879–10885 (1995).

14 Saric, M. & Scheibel, T. Two-in-One Spider Silk Protein with Combined Mechanical Features in All-Aqueous Spun Fibers. *Biomacromolecules* **24**, 1744–1750 (2023).

15 Chen, J., Hu, J., Sasaki, S. & Naka, K. Modular assembly of a conserved repetitive sequence in the spider eggcase silk: From gene to fiber. *ACS Biomaterials Science & Engineering* **4**, 2748–2757 (2018).

16 Peng, Q. *et al.* Recombinant spider silk from aqueous solutions via a bio-inspired microfluidic chip. *Scientific reports* **6**, 1–12 (2016).

17 Multiphysics, C. v. 5.6; COMSOL AB: Stockholm, Sweden, 2020. *URL www. comsol. com* (2022).

18 Müller, G. T. A. & Stokes, R. H. The mobility of the undissociated citric acid molecule in aqueous solution. *Transactions of the Faraday Society* **53**, 642–645 (1957).

19 Press, C. R. C. Inc., Cleveland, Ohio. *Handbook of Chemistry and Physics* (1977).
