# Supplementary material for: Replicating shear-mediated self-assembly of spider silk through microfluidics
Source: Nat Commun. 2024 Jan 15;15:527. doi: 10.1038/s41467-024-44733-1 (PMC10789810; doi:10.1038/s41467-024-44733-1)
Supplement: Supplementary file 3 — Description of Additional Supplementary Files [file 41467_2024_44733_MOESM3_ESM.pdf]

Description of Additional Supplementary Files

File Name: Supplementary Movie 1
Description: Demonstration of the experimental procedure for biomimetic microfluidic spinning under a negative control system.

File Name: Supplementary Movie 2
Description: 3D construction of NR12-C fiber formed inside the microfluidic device.

File Name: Supplementary Movie 3
Description: Biomimetic spinning of N-R12-C fiber inside the microfluidic device.

File Name: Supplementary Movie 4
Description: Biomimetic spinning of N-R12-C(xA) fiber inside the microfluidic device.
